# Titanium Cable Cerclage Increases the Load to Failure in Plate Osteosynthesis for Distal Femoral Fractures

**DOI:** 10.3390/medicina60091524

**Published:** 2024-09-19

**Authors:** Christopher Bliemel, Jakob Cornelius, Valerie Lehmann, Ludwig Oberkircher, Denis Visser, Bastian Pass, Steffen Ruchholtz, Martin Bäumlein

**Affiliations:** 1 Center for Orthopaedics and Trauma Surgery, University Hospital Giessen and Marburg, Baldingerstrasse, 35043 Marburg, Germany; jakob.cornelius@uk-gm.de (J.C.); valerielehmann@gmx.de (V.L.); visser@med.uni-marburg.de (D.V.); ruchholt@med.uni-marburg.de (S.R.); baeumlei@med.uni-marburg.de (M.B.); 2 Philipps-University Marburg, 35037 Marburg, Germany; oberkircher.ludwig@medizincampus.de; 3 Clinic for Trauma and Orthopaedic Surgery and Joint Replacement, Medical Campus Bodensee, 88048 Friedrichshafen, Germany; 4 Department of Orthopedic and Emergency Surgery, Alfried Krupp Hospital Essen, 45131 Essen, Germany; bastian.pass@krupp-krankenhaus.de

**Keywords:** distal femoral fracture, polyaxial angular stable plate osteosynthesis, titanium cable cerclage, biomechanical analysis, load to failure

## Abstract

*Background and Objectives:* The reduction of two-part oblique or spiral fractures of the distal femur using steel wire cerclage prior to plate osteosynthesis is a proven procedure. In addition to being useful in fracture reduction, wire cerclage was also shown to increase the stability of osteosynthesis. Nevertheless, metal corrosion and the allergenic potency of steel remain problematic disadvantages of this method. A biomechanical study was carried out to evaluate titanium cable cerclage as an alternative supplement for plate osteosynthesis of a distal femoral two-part fracture. *Materials and Methods:* An unstable AO/OTA 32-A2.3 fracture was created in eleven pairs of nonosteoporotic human cadaver femora. All the samples were treated with polyaxial angular stable plate osteosynthesis. One femur from each pair was randomly selected for an additional fracture fixation with multifilament titanium cable cerclage. Stepwise cyclic axial loading was applied in a load-to-failure mode using a servohydraulic testing machine. *Results:* All specimens (mean age: 80 years; range: 57–91 years) withstood a cycling force of at least 1800 N. With a mean load of 2982 N (95% CI: 2629–3335 N), the pressure forces resulting in osteosynthesis failure were significantly higher in specimens with an additional titanium cerclage (Group 1) than in samples that were solely treated with plate osteosynthesis (Group 2) at 2545 N (95% CI: 2257–2834 N) (*p* = 0.024). In both groups, cutting out the distal screws at the condyle region, resulting in shearing of the distal fragment proximal to the fracture line, was the most frequent cause of construct failure. Among the specimens assigned to Group 1, 36% exhibited a specific fracture pattern, namely, a fracture of the dorsal buttress above the cerclage. Analysis of axial stiffness (*p* = 0.286) and irreversible deformity of the specimens revealed no differences between the groups (*p* = 0.374). *Conclusion:* Titanium cable cerclage application, as a supplement to an angular stable plate, resulted in an increased load to failure. In terms of stability, the use of this adjunct for fracture fixation of supracondylar two-part oblique femoral fractures might, therefore, be an option, especially in patients who are sensitive to nickel.

## 1. Introduction

Distal femoral fractures have a bimodal distribution pattern, usually affecting either young and polytraumatized men or old-aged women in the sense of a geriatric low-energy trauma [1,2]. Surgical treatment of such fractures is challenging not only because of the patient collectives affected but also because of prevailing complex anatomical conditions. Performing polyaxial angular stable plate osteosynthesis is a proven procedure for the fixation of such fractures [3].

Not only in distal femur fractures but also in proximal femur fractures, even if a joint fracture occurs, a plate is not the only available devices of synthesis. It is possible to use intramedullary nailing in addition to the plate to strengthen the synthesis [4,5]. While Passias et al. proved that a combined nail–plate construct is a successful treatment alternative for acute management of distal femur fractures, Wilson et al. recently pointed out that there is currently no consensus on a standard of care, and little randomized evidence is available that directly compares intramedullary nails with plating [6,7].

Particularly in the case of two-part oblique or spiral fractures of the distal femur, a double-looped steel wire cerclage, as a supplement to plate osteosynthesis, facilitates fracture reduction and significantly increases the load to failure, as already shown in a biomechanical study [8].

Despite this advantage in terms of stability, steel cerclage provides some crucial drawbacks related to material texture. First, steel cerclage has the potential risk of increased corrosion due to friction, which may lead to encapsulation in connective tissue, increasing the sensitivity to infections and loosening [9]. Second, by using similar metals, namely a plate and a cerclage made of titanium, potential galvanic corrosion could be avoided. However, it should be said that it is still not clear whether galvanic corrosion of stainless wires and titanium plates has any negative effects at all [10]. Third, the allergenic potential of steel implants may not be forgotten since these implants contain up to 15% nickel [11].

In this context, nickel is the leading contact allergen in most industrialized countries worldwide. Even though in Europe the prevalence of nickel allergy has declined in some countries following the implementation of the EU Nickel Directive [12], its prevalence is still high, at approximately 8–19% in adults [13] and up to 10% in children and adolescents among the general population [14]. Although nickel allergy is less well investigated outside Europe, a persistently high prevalence was also observed in Asia and North America [15,16].

In this context, titanium cerclage overcomes the abovementioned disadvantages of metal corrosion and allergenic potency. Moreover, titanium cerclage provides a lower modulus of elasticity. Under comparable loads, pure titanium is almost twice as elastic as steel, offering the possibility of a more bone-like swinging, resulting in better fracture healing [17].

Already in other body regions, like the patella [18,19] or the subtrochanteric femur [20], titanium cerclage was shown to be an efficient supplement for fracture reduction and fixation.

**Figure 1:**
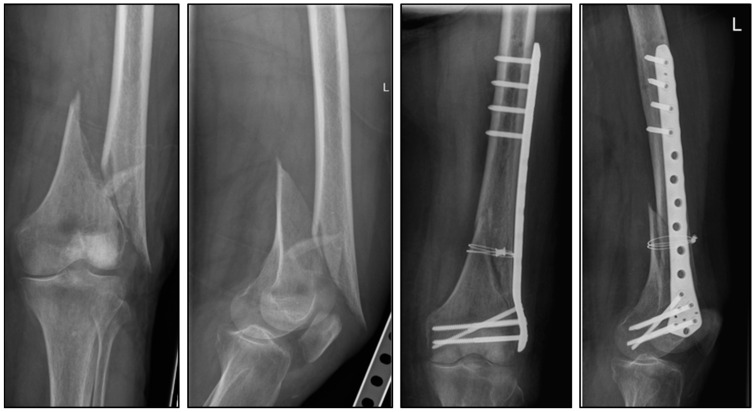
Clinical case of an 81-year-old female with a 33-A2.3 distal femoral fracture. Due to nickel allergy, the fracture was surgically treated with direct fracture repositioning using double-looped titanium cerclage and retrograde-inserted, polyaxially angular plate osteosynthesis.

To analyze how titanium cerclage acts as a supplement for plate osteosynthesis of a distal femoral two-part fracture, a biomechanical setup was used. It was suspected that titanium cerclage would enhance the compression at the fracture gap, leading to increased stability in the fracture region itself (Figure 1).

Due the results achieved with supplemental titanium cable cerclages in other body regions, the authors hypothesized that the load until failure would be greater for osteosynthesis constructs treated with additional titanium cerclage than for those treated with polyaxial angular stable plate osteosynthesis alone.

## 2. Materials and Methods

### 2.1. Samples

This biomechanical study was conducted on eleven pairs of adult human femora. The cadavers were embalmed with a solution consisting of 96% ethanol and <2% formaldehyde, as previously described [5,21]. The Institutes of Anatomy and Cell Biology at Philipps University Marburg made the femurs available because all donors had voluntarily agreed to the use of their bodies for research purposes before their death. The local authorities granted ethical approval (AZ 23/26 BO, 23 February 2023).

### 2.2. Evaluation of Bone Quality

After receiving the bones, conventional medial–lateral and anterior–posterior radiographs were taken of each femur to exclude pre-existing fractures or other pathologies. With the use of dual-energy X-ray absorptiometry (DXA) (Horizon (device), Inc. Hologic (company), Marlborough, MA, USA), the bone mineral density of the hip was measured for each femur.

### 2.3. Surgical Technique and Implants Used

A Noncontact Bridging plate for Distal Femur (NCB^®^-DF, Zimmer GmbH, Winterthur, Switzerland) was used for all specimens. The NCB^®^-DF plate is a polyaxial locking device that is available in left and right versions to ensure optimal adaptation to the lateral cortex of the femur. In this study, only NCB^®^-DF nine-hole plates, made of titanium alloy, were used. Two study surgeons (Christopher Bliemel and Martin Bäumlein) carried out all osteosyntheses on all specimens.

The NCB-DF plate was fixed at the distal end of the femur, 1.5 cm proximal to the lateral articular surface. For this purpose, the plate was attached with blunt bone reduction clamps. For shaft fixation, four bicortical, fully threaded cortical screws were inserted in the proximal part of the plate. At the distal part of the plate osteosynthesis, five monocortical, partially threaded cancellous screws were placed. In a laboratory setting, plate osteosynthesis was performed before creating an osteotomy gap due to a lack of stabilizing soft tissue for technical reasons.

### 2.4. Fracture Model

In accordance with a previously established fracture model, a supracondylar oblique femoral fracture was simulated (32-A2.3 according to the Arbeitsgemeinschaft für Osteosynthesefragen Foundation/Orthopaedic Trauma Association AO/OTA classification) after fixation of the NCB-DF plate to the femur [8]. Therefore, the individual condylar width was measured for each femur. Subsequently, an oblique osteotomy was created, starting at the level of one condylar length proximal to the joint line. The fracture line extended from the distal anterior cortex at an angle of 70° to the proximal posterior cortex of the femur. This type of osteotomy was chosen to prevent the shearing fracture fragments from resting on the laterally attached plate under the applied load during the test.

After osteotomy, the samples of each pair of femora were divided into two different groups. The randomization of the femora, whether the right or the left femur was treated with an additive cerclage, was conducted by coin toss.

**Figure 2:**
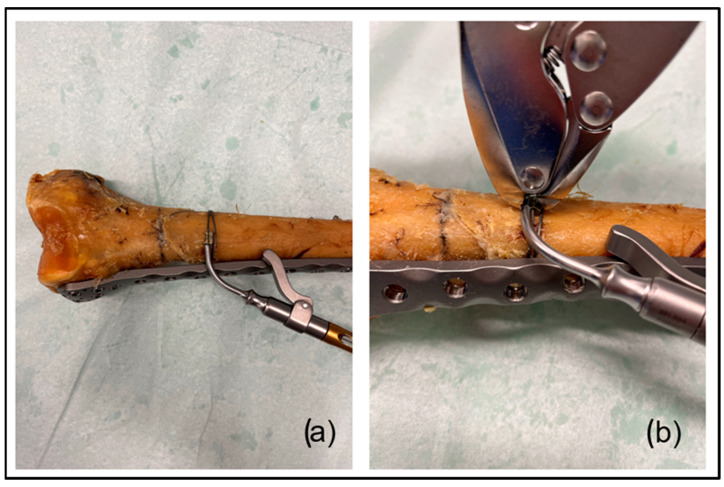
Images illustrating the application of the titanium cerclage. Photograph (**a**) demonstrates the use of the cable tensioner. After the desired tension was achieved (200 N), a cable crimper was used to tighten the cerclage (photograph (**b**)).

Thereafter, a double-looped titanium cable cerclage (DePuy Synthes, West Chester, PA, USA) was attached to all specimens assigned to Group 1. The cerclage was positioned in the middle of the oblique fracture. The cable cerclage used has a multifilament structure consisting of a central bundle with nineteen strands and eight outer bundles with seven strands [22]. In order to provide comparable results to previous research on this topic [8], in the present study, only 1.0 mm titanium cable cerclages, which are made of titanium alloy, were used. As recommended by the manufacturer, a cable tensioner was used. To provide reproducible results among the specimens and with previous literature, the applied tension was set at 200 N [8,23]. After the desired tension was achieved, the cable crimp was precisely deformed using a cable crimper. The cable was cut thereafter using the intended cable cutter. Figure 2 shows the tensioning of the titanium cable cerclage and the crimping of the cerclage, as they represent the most crucial steps of the cable application procedure.

**Figure 3:**
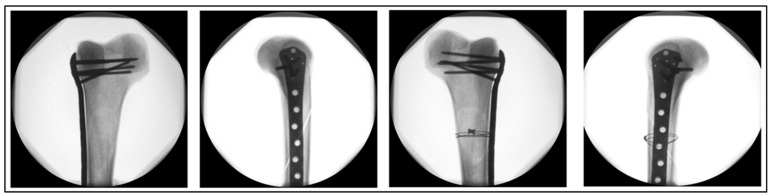
Typical postoperative X-rays of a sample pair illustrating an anatomical fracture reduction with plate osteosynthesis in the left specimen (Group 2) and plate fixation with an additional titanium cable cerclage in the right specimen (Group 1).

All femora assigned to Group 2 did not receive a wire cerclage. Figure 3 illustrates the fracture fixation in the two groups, either with an additional supply of wire cerclage (Group 1) or not (Group 2).

### 2.5. Biomechanical Cyclic Loading

**Figure 4:**
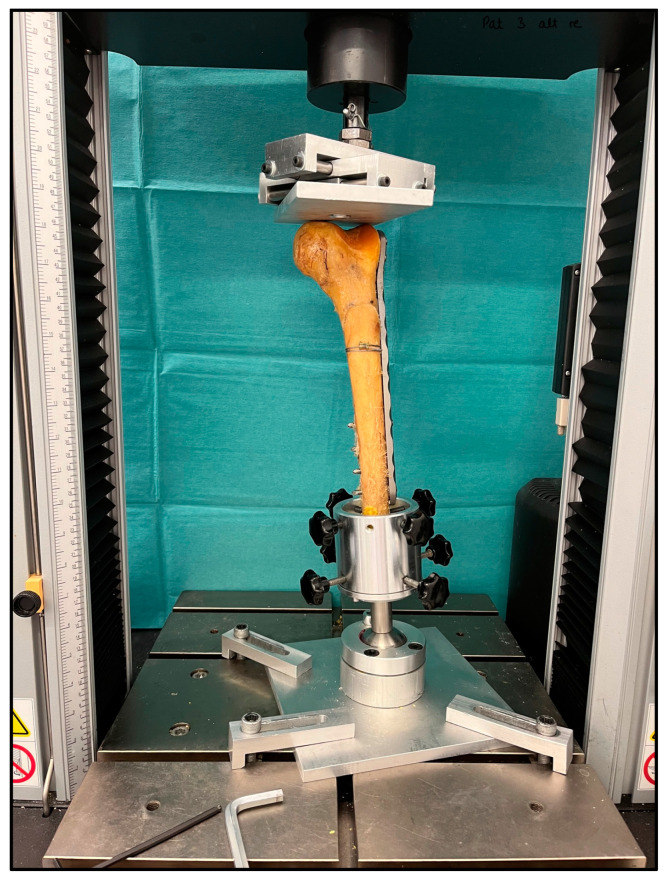
Photograph demonstrating the test setup with an upside-down position of the femur in an Instron 5566 servohydraulic testing machine. The load cell introduced the set force via a custom-made metal plate over the condyles along the mechanical leg axis.

All specimens were shortened 6 cm proximal to the plate osteosynthesis site. Subsequently, the proximal part of each femur was embedded in special pots using self-hardening two-component resin (Technovit 3040, Heraeus, Wehrheim, Germany). With specially designed fixtures, the specimens were positioned upside down in a universal servohydraulic testing machine (Instron 5566, Instron Cor., Darmstadt, Germany). The construct was loaded axially via a metal plate that can be moved in two directions (anterior–posterior and medial–lateral). To allow loading along the mechanical axis of the leg, the condyles of the femur were positioned parallel to the ground (Figure 4).

The loading tests were carried out analogously to a previous test by our working group [8]. To densify the samples and increase bearing rigidity, a preload of 100 N was applied to each femur. Subsequently, each femur was loaded cyclically according to a standardized protocol. The test began with 500 cycles at 800 N. Thereafter, the load was continuously increased in increments of 200 N every 500 cycles until failure of the fixation device was reached. Construct failure was determined as a sudden loss of measured force (>30%) or a severe deformation of the osteosynthesis construct (>20 mm). The test was carried out in a path-controlled mode.

### 2.6. Data Acquisition and Statistical Evaluation

The instrument-specific user software for the Instron 5566 testing machine (Bluehill 2, Instron corporation, Norwood, MA, USA) recorded the loading (N), deformation (mm), and number of cycles at 100 ms intervals.

The plastic deformation, as a measure of irreversible deformity under the influence of applied force, was calculated by subtracting the initial construct height from the deformity that existed after the end point was reached once the load had been taken away. The stiffness of the samples was calculated from the compression deformation caused by the applied load.

Grounded on the load-to-failure values reported in previous biomechanical studies dealing with distal femoral fractures, an a priori power analysis was conducted [5,8,24]. Based on a statistical power of 0.80 and an alpha error of 0.05, a sample size of eleven pairs of femora was assumed. Failure of osteosynthesis in 85% of all osteosyntheses with angle-stable plate osteosynthesis and in up to 30% of the specimens with combined plate and wire cerclage osteosynthesis at a load of 2800 N was defined as a clinically relevant difference.

For statistical analysis, IBM SPSS statistics 27 was use (Statistical Package for the Social Science, IBM Cooperation, Armonk, New York, NY, USA). Due to the limited number of samples, for data analysis the Wilcoxon rank-sum test (nonparametric test) for paired samples was applied. For nominal scaled variables, the chi-square test was used. Statistical significance was assumed at *p* < 0.05.

## 3. Results

### 3.1. Specimen Characteristics

The samples available for this biomechanical study were obtained from two female and nine male body donors. These body donors had an average age of 80 years (range 57–91 years). Preoperative conventional anterior–posterior and medial–lateral radiographs of the femora provided no evidence of pre-existing osteolysis, bone tumors, or fractures in any of the examined specimens. DXA confirmed intact bone quality in all of the specimens available for this study, with a mean T score of −1.20 (CI: −1.68 to −0.73). Furthermore, bone mineral density was comparable in all of the matched pairs of cadaver femora (*p* = 0.219).

### 3.2. Cyclic Testing

**Figure 5:**
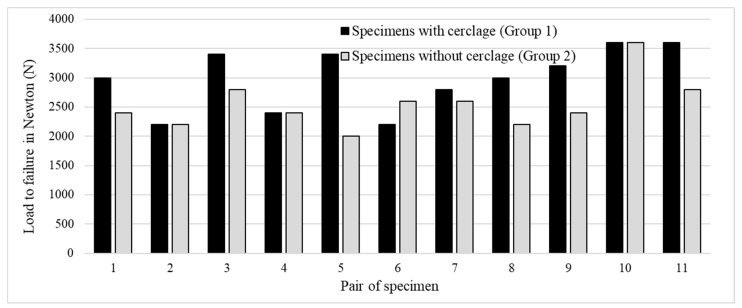
Depiction of the load to failure in the eleven pairs of examined specimens. The black bars illustrate the samples that were stabilized using plate osteosynthesis and additional titanium cerclage. The samples treated exclusively with plate osteosynthesis are represented by gray bars.

All specimens at least withstood a cycling force of 1800 N. The mean compressive load leading to failure in specimens with an additional titanium cerclage (Group 1) was 2982 N (95 CI: 2629–3335 N). At a mean load of 2527 N (95 CI: 2237–2827 N), the cerclage agent in that group tore. In Group 2, for the specimens without cerclage, the mean compressive load to failure was 2545 N (95% CI: 2257–2834 N) (Figure 5).

The difference in load to failure between specimens with an additional titanium cerclage (Group 1) and those specimens without cerclage (Group 2) reached statistical significance (*p* = 0.024).

### 3.3. Analysis of the Samples When They Reached the Abortion Criterion

After cyclic loading, all specimens were examined clinically and radiologically to evaluate their fracture behavior when they reached the failure criterion. In Group 1, in six out of eleven specimens, the distal screws were cut out of the condyle region, resulting in shearing of the distal fragment proximal to the fracture line. Two times, the distal screws were tilted without being cut out of the condylar block. Multifragmentary shaft fracture and irreversible deformation of the plate occurred once each in Group 1. A tear of the cerclage occurred in nine out of the eleven specimens. Interestingly, there was never failure in the area of the cerclage lock, but there was always a tear in the cerclage itself. In Group 1, four out of the eleven specimens exhibited a specific fracture pattern, namely, a fracture of the dorsal buttress above the cerclage (Table 1).

**Figure 6:**
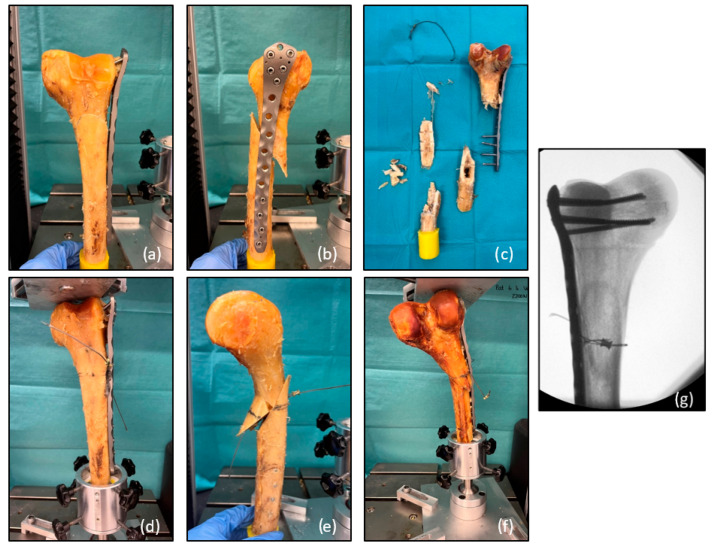
Images illustrating the different types of osteosynthesis failure. Photographs (**a**,**b**) demonstrate the most common type of failure in Group 2, with cutting of the distal screws out of the condyle region leading to a shear of the condyles along the fracture line toward the proximal region. Picture (**c**) illustrates a multifragmentary shaft fracture with an additional tear of the cerclage in a specimen in Group 1. Photographs (**d**,**e**) show the most common fracture pattern in Group 1, corresponding to the fracture pattern shown in pictures (**a**,**b**). In 36% of the patients in Group 1, a fracture of the dorsal buttress proximal to the cerclage occurred (with additional cerclage in this patient). Photograph (**f**) shows irreversible deformation of the plate with an additional tear of the cerclage. The X-ray image in (**g**) shows the tilt of the distal screws without cutting out of the condylar block. This type of failure occurred in both groups.

In Group 2, without titanium cerclage, the test was aborted in seven out of eleven cases due to cutting out of the condylar screws combined with shearing of the distal fragment along the fracture line. Three times a tilt of the distal screws without cutting out of the condylar block occurred. Additionally, one multifragmentary fracture was observed in Group 2 (Table 1). The photographs shown in Figure 6 illustrate the different fracture patterns that occurred in both groups.

### 3.4. Comparison of the Axial Stiffness and Plastic Deformation of the Osteosynthesis Construct at a Load of 1800 N

To determine the bottom of the different failure loads, a stiffness analysis of the different osteosynthesis constructs was conducted. A comparison of both constructs was carried out at a load of 1800 N since this was the last time point when all samples in both groups were still intact.

The average stiffness of the osteosynthesis construct in Group 1 was 2.05 N/m (95% CI: 1.64–2.47 N/m). In Group 2, with solely plate osteosynthesis, the mean value was 2.48 N/m (95% CI: 1.83–3.13 N/m). This difference in axial stiffness at a load of 1800 N was not significant (*p* = 0.286).

**Figure 7:**
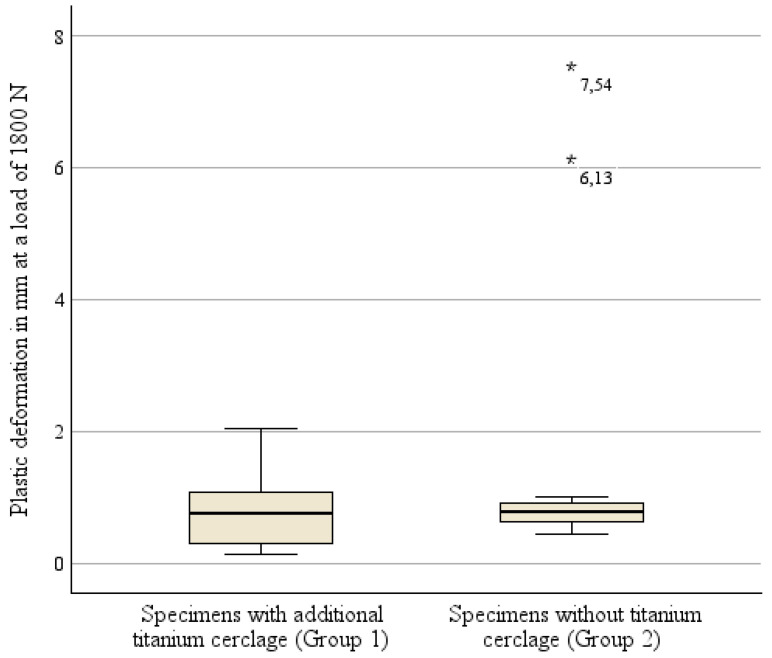
Boxplot illustration of irreversible deformation of both types of osteosynthesis at a load of 1800 N.

Additionally, plastic deformation of the osteosynthesis construct was analyzed at a load of 1800 N. As shown in Figure 5, a lower variability in plastic deformation was revealed if osteosynthesis was conducted with an additional titanium cerclage (mean 0.82 mm; 95% CI 0.39–1.25 mm) compared to that of the specimens treated without cerclage (mean 1.83 mm; 95% CI 0.15–3.51 mm). Figure 7 additionally demonstrates the two statistical outliers in Group 2 with plastic deformations of 6.13 mm and 7.45 mm at that loading strength. Nevertheless, this difference in plastic deformation between the two osteosynthesis groups failed to reach statistical significance (*p* = 0.374).

## 4. Discussion

Treatment of distal femur fractures remains a challenge, even in modern trauma and orthopedic surgery. In this biomechanical study, the potential of a titanium cable cerclage, in addition to an angular stable plate osteosynthesis for the fixation of a two-part femur fracture was investigated. Compared with plate osteosynthesis alone, titanium cable cerclage, which is applied in addition to plate osteosynthesis, significantly increased the load to failure. The application of such cerclage did not affect the axial stiffness or plastic deformation of the samples.

Having conducted a clinical study on 56 patients with displaced two-part distal femur fractures, Lee et al. showed that percutaneous-cerclage-wiring-assisted reduction and the minimally invasive plate osteosynthesis (MIPO) technique resulted in better reduction and faster union time in elderly patients [25]. Similar results have been reported by Apivatthakakul et al., who used percutaneous cerclage wiring in patients with Vancouver type B1 fractures treated with percutaneous cerclage wiring for fracture reduction and maintenance of reduction with MIPO [26].

Apart from these positive effects in terms of fracture reduction and healing time, a previous biomechanical examination by our own study group demonstrated the failure load-increasing effect of steel wire cerclage as a supplement to plate osteosynthesis for the fixation of supracondylar femoral shaft fractures [8]. The use of such steel cerclage in combination with a plate made of titanium alloy nevertheless represents a potential drawback of this former study, not only because of the allergenic potential of the steel implants themselves but also because of the mixture of materials, which is controversial [27,28,29].

As allergic contact dermatitis to nickel is common and mediated through a type-IV allergic delayed cell-mediated response, steel cerclage is not suitable for every patient [30]. Repeated or prolonged exposure to specific compounds such as steel cerclages, can sensitize T cells to activate and induce an immune response at the sites of irritation [31]. Dermatitis, systemic skin rashes starting at the surgical site and spreading [32], and impaired wound healing, swelling, pain, and inflammation at the implant site, mimicking infection, can be possible consequences [33]. Although the lines presented above, regarding the T-cell-mediated immune system are only correct in living patients, this aspect is of only minor importance in a biomechanical study on cadaveric femurs. Nevertheless, titanium implants as alternative devices are known for their biocompatibility, corrosion resistance, and mechanical properties close to those of cortical bone [34].

The results of the present biomechanical study demonstrated that in combination with plate osteosynthesis, titanium cable cerclage, as an alternative to steel cerclage, led to a significantly increased load to failure compared to plate osteosynthesis alone. As a clinically relevant difference, failure of osteosynthesis in 85% of all osteosyntheses with angle-stable plate osteosynthesis and up to 30% of the specimens with combined plate and wire cerclage osteosynthesis at a load of 2800 N was defined. This assumption is based on in vivo biomechanical loading analyses of femur and hip contact forces during gait patterns published in the literature [35]. In this context, Bergmann et al. could prove that forces affecting the lower limb increase to as high as 240% of body weight during normal walking and up to 250 and 260% of body weight when going upstairs and downstairs, respectively [35].

To the best of the authors’ knowledge, a direct comparison of the advantages and disadvantages of titanium versus steel cerclage in addition to plate osteosynthesis for distal femur fracture has not been provided in the current literature. Hägerich et al. analyzed the performances of four different types of cerclages in biomechanical comparative stetting. Their results confirm the findings of the present study, as they demonstrated that titanium cable cerclages were among those types of cerclages that withstood the highest loads until failure [9]. Among the analyzed cerclages in their study, titanium cable cerclage also provided the highest construct stiffness. In contrast, axial stiffness, as a modulus of elasticity of the osteosynthesis constructs, remained unaffected in our analysis. A possible explanation for this difference could be that Hägerich et al. analyzed cerclage properties as independent osteosynthesis, whereas in the present study, titanium cerclage was tested as a supplement to angular stable plate osteosynthesis.

A previous investigation by our group confirmed that plastic deformation of the osteosynthesis construct decreased if a supracondylar femur fracture was fixed with steel cerclage as a supplement to an angular stable plate [8]. In the present study, only insignificant differences in irreversible deformation of the osteosynthesis construct were observed. Even though titanium implants are less resistant to initial plastic deformation than steel implants are [36], it must be assumed that these effects were concealed in the present study by the load properties of plate osteosynthesis. Future investigations might include a comparative evaluation between cerclage, plate alone, and the use of compression screws to demonstrate the possible superiority of one technique over the other not only in terms of stability but also concerning natural behaviors as indicated by construct stiffness.

## 5. Limitations

Although the present biomechanical study attempted to overcome the material-induced drawbacks of previous studies on this topic, some limitations remain. First, the use of human cadaveric bones embalmed with formaldehyde must be mentioned, as there are some controversies in the current literature concerning their authenticity. While Hammer et al. [37] and Unger et al. [38] pointed out organic changes through the preservation process in cadaveric bones, the findings of Topp et al. were able to prove that embalmed femora are comparable to fresh frozen bone in terms of the extraction forces of cancellous and cortical screws and axial load until failure [39]. Another possible limitation concerns the in vitro setup used for this biomechanical study, which only tested axial loading of the specimens. These restrictions in loading conditions, in which bending or torsional loading were not considered, could have influenced the results with regard not only to the failure load but also to the failure mode. Future research directions might include finite element analyses to address these limitations. Finally, with eleven pairs of femurs, the number of specimens available for the test was manageable. While sample limitation is always a concern in biomechanical studies among the existing studies on this topic, the present study is the largest to date, which definitely strengthens the value of this present investigation.

## 6. Conclusions

The application of titanium cable cerclage, as a supplement to an angular stable plate, resulted in an increased load to failure. In terms of stability, the use of this adjunct for fracture fixation of supracondylar two-part oblique femoral fractures might, therefore, be an option, especially in patients who are sensitive to nickel.

## Figures and Tables

**Table 1:** Fracture behavior when reaching the termination criteria. Multiple options were possible.

Failure Mode	Group 1—Plate Osteosynthesis with an Additional Cerclage (*n* = 11)	Group 2—Plate Osteosynthesis without a Cerclage (*n* = 11)	*p*-Value
Cutting out of the condylar screws with shearing of the distal fragment along the fracture line	6 (55%)	7 (64%)	0.665
Tilt of the distal screws without cutting out of the condylar block	2 (18%)	3 (27%)	0.611
Multifragmentary fracture of the femur shaft	1 (9%)	1 (9%)	1.000
Irreversible bowing of the plate	1 (9%)	0 (0%)	0.306
Fracture of the dorsal buttress proximal to the cerclage	4 (36%)	n.a.	n.a.
Tear of the cerclage	9 (82%)	n.a.	n.a.

n.a. = not applicable.

## Data Availability

The data and materials are available from the corresponding author upon reasonable request.

## Abbreviations

AO/OTA	Arbeitsgemeinschaft für Osteosynthesefragen/Orthopedic Trauma Association
CI	confidence interval
DXA	dual-energy X-ray absorptiometry
N	Newton
n.a.	not applicable
NCB^®^-DF	Noncontact Bridging plate for Distal Femur
m	meter
mm	millimeter
